# ASPECTS as a surrogate marker of core-perfusion mismatch in late-window large vessel occlusion stroke

**DOI:** 10.1093/esj/aakag065

**Published:** 2026-06-08

**Authors:** Pierre Seners, Nicole Yuen, Davide Strambo, Emmanuel Carrera, Mirjam R Heldner, Jean-Marc Olivot, Michael Mlynash, Jeremy J Heit, Elisabeth Dirren, Patrik Michel, Christophe Cognard, Julien Savatovsky, William Almiri, Maarten G Lansberg, Gregory W Albers

**Affiliations:** Neurology Department, Hôpital Fondation A. de Rothschild, FHU NeuroVasc 2030, Paris, France; Université Paris Cité, Institute of Psychiatry and Neuroscience of Paris (IPNP), INSERM U1266, Team Turc, Paris 75014, France; Stroke-Link F-CRIN Research Network, Lille, France; Stanford Stroke Center, Stanford, CA, United States; Stroke Center, Neurology Service, Lausanne University Hospital and University of Lausanne, Lausanne, Switzerland; Neurology Department, Geneva University Hospitals, Geneva, Switzerland; Neurology Department, Inselspital, Bern University Hospital, University of Bern, Bern, Switzerland; Stroke-Link F-CRIN Research Network, Lille, France; Acute Stroke Unit, Hôpital Pierre-Paul Riquet, CHU Toulouse and CIC 1436, Toulouse University, Inserm, UPS, Toulouse, France; Stanford Stroke Center, Stanford, CA, United States; Radiology Department, Stanford University, Palo Alto, CA, United States; Neurology Department, Geneva University Hospitals, Geneva, Switzerland; Stroke Center, Neurology Service, Lausanne University Hospital and University of Lausanne, Lausanne, Switzerland; Stroke-Link F-CRIN Research Network, Lille, France; Neuroradiology Department, Toulouse University Hospital, Toulouse, France; Radiology Department, Hôpital Fondation A. de Rothschild, Paris, France; Department of Diagnostic and Interventional Neuroradiology, University Hospital and University of Bern, Bern, Switzerland; Stanford Stroke Center, Stanford, CA, United States; Stanford Stroke Center, Stanford, CA, United States

**Keywords:** mismatch, stroke, thrombolysis, thrombectomy

## Abstract

**Introduction:**

In acute stroke due to a large vessel occlusion (AIS-LVO), identifying a core-perfusion mismatch guides reperfusion therapy in extended time window, but perfusion imaging is not universally available. We investigated whether the Alberta Stroke Program Early CT Score (ASPECTS) is associated with core-perfusion mismatch and could serve as a surrogate imaging marker.

**Patients and methods:**

We retrospectively analysed a large multicentre international unselected cohort of consecutive AIS-LVO patients imaged between 4.5 and 24 h from last time seen well, with baseline CT- or MR-perfusion imaging. Core-perfusion mismatch was defined as a mismatch volume ≥ 15 mL and a mismatch ratio ≥ 1.8 using automated software. CT and MRI cohorts were analysed separately.

**Results:**

Among 531 included patients, 182 underwent CT and 349 MRI. Core-perfusion mismatch was present in 86% of CT patients and 62% of MRI patients. Alberta Stroke Program Early CT Score was strongly associated with mismatch in both cohorts (*P* < .001). In the MRI cohort, ASPECTS predicted mismatch with an area under the receiver operating characteristic curve (AUC) of 0.843 (95% CI, 0.797–0.890), with an optimal cutoff ≥ 6, yielding sensitivity 0.87, specificity 0.72, positive predictive value (PPV) 0.84 and negative predictive value (NPV) 0.77. In the CT cohort, the AUC was 0.801 (95% CI, 0.698–0.904), with an optimal cutoff ≥ 7, yielding sensitivity 0.74, specificity 0.81, PPV 0.96 and NPV 0.34.

**Conclusion:**

The high PPV of ASPECTS ≥ 6 on MRI and ASPECTS ≥ 7 on CT supports their potential use as pragmatic enrichment criteria for patient selection in future late-window AIS-LVO trials, when perfusion imaging is unavailable, as surrogate markers of salvageable tissue.

## Graphical Abstract

**Graphical Abstract:**
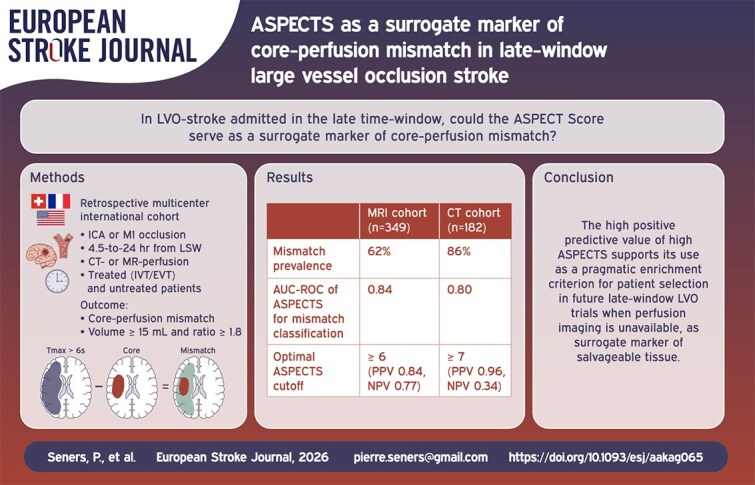

## Teaser

Stephanie Kemp, Mikael Mazighi, Michel Piotin, Guillaume Bellanger, Thomas Tourdias, Anne-Christine Januel, Fabrice Bonneville, Nicolas Raposo, Alain Viguier, Lionel Calvière, Paolo Machi, Gianmarco Bernava, Manuela Buehrer, Fanny Simonnet, Sarah J. Snyder, Jose Bernardo Escribano-Paredes, Alexander Salerno, Michael Obadia, Igor Sibon.

## Introduction

In acute ischaemic stroke due to a large vessel occlusion (AIS-LVO), CT- or MRI-perfusion imaging can estimate the amount of salvageable ischaemic tissue by assessing the mismatch between severely hypoperfused tissue and the irreversibly injured infarct core (ie, the core-perfusion mismatch).^1^^,^^2^ Identification of a core-perfusion mismatch is recommended by international guidelines to guide intravenous thrombolysis (IVT) or endovascular therapy (EVT) in the extended time window (>4.5 h for IVT and > 6 h for EVT),^3–5^ as patients meeting these criteria derive substantial benefit from reperfusion therapies, whereas no substantial treatment benefit was observed in patients without mismatch or in trials that did not use mismatch-based selection.^6–9^ However, despite major progress made in the last decade, the acquisition and automatic fast post-processing remains limited in some regions and care settings, particularly in non-comprehensive stroke centres.^10^^,^^11^ Identifying reliable surrogate imaging biomarkers of the core-perfusion mismatch is therefore crucial, as it could pave the way for future trials aimed at expanding access to these effective therapies for patients in whom direct assessment of the core-perfusion mismatch is unavailable.

In this large, multicentre international cohort of unselected AIS-LVO patients who underwent baseline CT- or MRI-perfusion imaging beyond 4.5 h from the last time seen well, we aimed to investigate the relationship between core-perfusion mismatch and the Alberta Stroke Program Early CT Score (ASPECTS), a widely used, semi-quantitative visual rating scale that assesses the extent of the infarct core on baseline imaging.

## Methods

Our analysis was reported following the Strengthening the Reporting of Observational Studies in Epidemiology criteria for observational studies.^12^

### Study design and data sources

The Mismatch Prevalence Collaboration^13^ retrospectively collected the data from consecutive AIS-LVO patients extracted from (1) the prospective registries of 5 academic comprehensive stroke centres with systematic use of CT- or MR-perfusion imaging that include both IVT- or EVT-treated and untreated patients (Rothschild Foundation Hospital, Paris, France; Stanford Hospital, Palo Alto, CA; Geneva University Hospital, Switzerland; Lausanne University Hospital, Switzerland and Bern University Hospital, Switzerland), and (2) one EVT trial where CT- or MR-perfusion imaging was acquired per protocol but treatment decisions were made blinded to its results (FRAME).^14^ Inclusion dates were from September 2020 to June 2022 in Paris, January 2015 to June 2022 in Stanford, January 2018 to December 2021 in Geneva, January 2016 to December 2021 in Lausanne, January 2018 to May 2019 in Bern and January 2017 to February 2019 in FRAME. The following inclusion criteria were applied: (1) baseline MRI or CT, including perfusion, performed between 4.5 and 24 h from the last time seen well showing intracranial internal carotid artery (ICA) or first segment of the middle cerebral artery (M1) occlusion on MRA or CTA; and (2) baseline NIHSS score > 5. Patients with technically inadequate images were excluded.

### Clinical and radiological data

Clinical variables routinely recorded in the acute stroke setting were collected.^13^ The following radiological variables were collected: (1) ASPECTS, on noncontrast CT or diffusion-weighted imaging (DWI); (2) core volume, defined on DWI as an apparent diffusion coefficient < 620 × 10^−6^ mm^2^/s or on CT perfusion as relative cerebral blood flow < 30% of normal brain; (3) time-to-maximum (*T*max) > 6 s volume; (4) mismatch volume, defined as *T*max > 6 s volume—core volume; (5) mismatch ratio, defined as *T*max > 6 s volume/core volume; and (6) occlusion site on MR or CT angiography, dichotomised into intracranial ICA and M1. All DWI and perfusion imaging were processed by RAPID software (iSchemaView, MenloPark, CA). One reader centrally reviewed the imaging ASPECTS, core and *T*max maps at 4 of the 6 sites, with manual correction of core/*T*max artefacts when needed. In the remaining 2 centres, image review was performed locally by a dedicated reader at each site. Alberta Stroke Program Early CT Score was assessed while blinded to perfusion imaging data.

The primary outcome was the presence of a core-perfusion mismatch, defined as mismatch volume ≥ 15 mL and mismatch ratio ≥ 1.8.^15^ Two alternative core-perfusion mismatch definitions were also studied: (1) mismatch volume ≥ 15 mL and mismatch ratio ≥ 1.8 and core volume < 70 mL,^16–18^ and (2) mismatch volume > 10 mL and mismatch ratio > 1.2 and core volume < 70 mL.^19^^,^^20^

### Statistical analysis

The CT and MRI cohorts were analysed separately, given the well-known intrinsic differences in ASPECTS scoring and in the estimation of core and *T*max volumes between imaging modalities. Continuous variables were described as median (IQR), and categorical variables as counts and percentages. In each cohort, the best ASPECTS cutoff to classify core-perfusion mismatch was identified using the Youden index (ie, the cutoff that maximises sensitivity and specificity) in receiver operating characteristic (ROC) curve analysis. The diagnostic accuracy (sensitivity, specificity, negative and positive predictive values) of several ASPECTS cutoffs for core-perfusion mismatch identification were calculated. Among included patients, there were no missing ASPECTS or core-perfusion mismatch data. Statistical analyses were performed using SPSS version 30.0 (IBM, Armonk, NY). Two-tailed *P* < .05 was considered significant.

### Ethical approval and data availability

The research was approved by the Rothschild Foundation Hospital review board (IRB 00012801, under the study number CE_20220726_8_PSS). In FRAME, each participant signed a written informed consent. In the other centres, the requirement for written informed consent was waived because this study only implied retrospective analysis of anonymised data collected as part of routine care. The data supporting the study findings are available from the corresponding author on reasonable request.

## Results

### Patient cohort

During the study period, 1559 patients with an ICA or M1 occlusion were admitted to the participating centres. Among them, 1028 patients were excluded for the following reasons: NIHSS score < 6 (*n* = 125), admission within 4.5 h from the last time seen well (*n* = 835), perfusion imaging not performed (*n* = 35) or perfusion imaging of inadequate quality (*n* = 33), leaving 531 patients for the final analysis. Patients excluded because perfusion imaging was unavailable or of inadequate quality, but who otherwise met all inclusion criteria (*n* = 68), had baseline characteristics similar to those of included patients, except for a higher prevalence of ICA occlusions (Table S1).

Among the 531 included patients, 182 patients had CT and 349 had MRI. The baseline clinical and radiologic characteristics in the CT and MRI cohorts are shown in Table 1. Compared with patients in the MRI cohort, those in the CT cohort were older, less often male, more likely to have an unwitnessed stroke onset, and had longer last-seen-well-to-imaging times. They also had higher ASPECTS values, smaller core volumes, but larger *T*max > 6-s and mismatch volumes. The prevalence of core-perfusion mismatch was 86% (156/182) in the CT cohort and 62% (216/349) in the MRI cohort.

**Table 1:** Main characteristics of the patients in the CT and MRI cohorts.

	CT cohort, *n* = 182	MRI cohort, *n* = 349	*P*-value
**Age, years**	79 (67–87)	72 (60–82)	<.01
**Male**	69 (38)	172 (49)	.01
**Unwitnessed stroke onset**	139 (76)	186 (53)	<.01
**NIHSS score**	19 (12–23)	17 (13–22)	.17
**Onset-to-imaging time, h**	10.9 (6.7–15.2)	8.6 (6.0–13.1)	<.01
**Occlusion site**			.75
** ICA**	59 (32)	118 (34)	
** M1**	123 (68)	231 (66)	
**ASPECT score**	8 (6–9)	6 (4–8)	<.01
** 0–5**	45 (25)	124 (36)	.01
** 6–10**	137 (75)	225 (64)	
**Core volume, mL**	16 (0–52)	29 (13–88)	<.01
***T*max > 6 s volume, mL**	143 (99–195)	105 (65–148)	<.01
**Mismatch volume, mL**	108 (71–150)	47 (16–82)	<.01
**Core-perfusion mismatch**	156 (86)	216 (62)	<.01
**EVT performed**	127 (70)	252 (72)	.56

Abbreviations: ICA = intracranial internal carotid artery; ASPECTS = Alberta Stroke Program Early CT Score; *T*max = time to maximum; M1 = first segment of the middle cerebral artery.

### Prevalence of core-perfusion mismatch according to ASPECT score

#### MRI cohort

**Figure 1:**
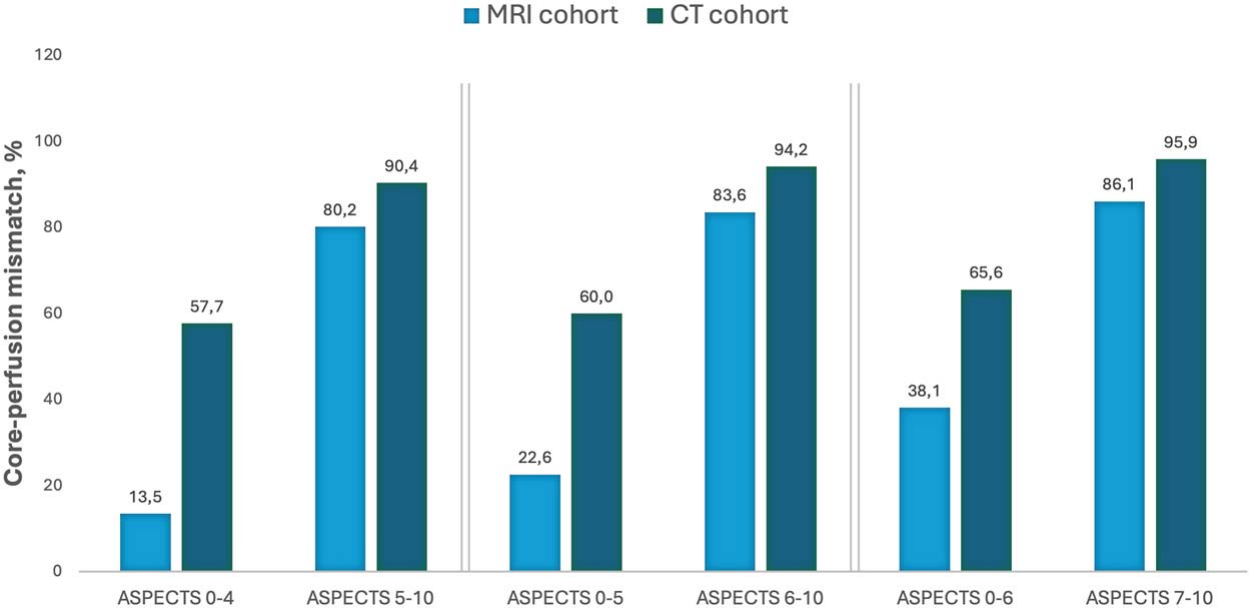
Prevalence of core-perfusion mismatch across different ASPECTS cutoffs. Abbreviation: ASPECTS = Alberta Stroke Program Early CT Score.

Alberta Stroke Program Early CT Score was strongly associated with the presence of a core-perfusion mismatch: median (IQR) ASPECTS was 7 (6–8) in patients with a mismatch and 4 (1–6) in those without (*P* < .001). The area under the ROC curve of ASPECTS for predicting core-perfusion mismatch was 0.843 (95% CI, 0.797–0.890). The optimal ASPECTS cutoff to classify core-perfusion mismatch was 6. In patients with ASPECTS ≥ 6, the sensitivity, specificity, positive and negative predictive values were 0.87, 0.72, 0.84 and 0.77, respectively. The diagnostic performance of several ASPECTS cutoffs for identifying core-perfusion mismatch is shown in Table 2 and Figure 1. The results were consistent in the witnessed and unwitnessed stroke-onset subgroups (Table 3). Similar results were observed in the sensitivity analyses using the alternative core-perfusion mismatch definitions (Tables S2 and S3).

**Table 2:** Diagnostic accuracy of ASPECTS cutoffs for core-perfusion mismatch.

	Sensitivity (95% CI)	Specificity (95% CI)	Positive predictive value (95% CI)	Negative predictive value (95% CI)
**MRI cohort**				
** ASPECTS ≥ 4**	0.96 (0.93–0.98)	0.49 (0.40–0.58)	0.75 (0.70–0.80)	0.89 (0.80–0.95)
** ASPECTS ≥ 5**	0.94 (0.90–0.97)	0.62 (0.54–0.71)	0.80 (0.75–0.85)	0.86 (0.78–0.93)
** ASPECTS ≥ 6**	0.87 (0.82–0.91)	0.72 (0.64–0.80)	0.84 (0.78–0.88)	0.77 (0.69–0.84)
** ASPECTS ≥ 7**	0.69 (0.62–0.75)	0.82 (0.74–0.88)	0.86 (0.80–0.91)	0.62 (0.54–0.69)
** ASPECTS ≥ 8**	0.46 (0.39–0.53)	0.87 (0.80–0.92)	0.85 (0.78–0.91)	0.50 (0.43–0.56)
**CT cohort**				
** ASPECTS ≥ 4**	0.94 (0.89–0.97)	0.35 (0.17–0.56)	0.90 (0.84–0.94)	0.47 (0.24–0.71)
** ASPECTS ≥ 5**	0.90 (0.85–0.95)	0.42 (0.23–0.63)	0.90 (0.85–0.95)	0.42 (0.23–0.63)
** ASPECTS ≥ 6**	0.83 (0.76–0.88)	0.69 (0.48–0.86)	0.94 (0.89–0.97)	0.40 (0.26–0.56)
** ASPECTS ≥ 7**	0.74 (0.67–0.81)	0.81 (0.61–0.93)	0.96 (0.91–0.99)	0.34 (0.23–0.48)
** ASPECTS ≥ 8**	0.56 (0.48–0.64)	0.85 (0.65–0.96)	0.96 (0.89–0.99)	0.24 (0.16–0.35)

Abbreviation: ASPECTS = Alberta Stroke Program Early CT Score.

**Table 3:** Diagnostic accuracy of the optimal ASPECTS cutoff for core-perfusion mismatch in witnessed and unwitnessed stroke-onset subgroups.

	Sensitivity (95% CI)	Specificity (95% CI)	Positive predictive value (95% CI)	Negative predictive value (95% CI)
**MRI cohort (ASPECTS ≥ 6)**				
** Overall population (*n* = 349)**	0.87 (0.82–0.91)	0.72 (0.64–0.80)	0.84 (0.78–0.88)	0.77 (0.69–0.84)
** Witnessed onset (*n* = 163)**	0.89 (0.82–0.95)	0.78 (0.66–0.88)	0.88 (0.80–0.93)	0.81 (0.69–0.90)
** Unwitnessed onset (*n* = 186)**	0.85 (0.77–0.91)	0.67 (0.55–0.78)	0.80 (0.72–0.87)	0.74 (0.62–0.84)
**CT cohort (ASPECTS ≥ 7)**				
** Overall population (*n* = 182)**	0.74 (0.67–0.81)	0.81 (0.61–0.93)	0.96 (0.91–0.99)	0.34 (0.23–0.48)
** Witnessed onset (*n* = 43)**	0.76 (0.59–0.88)	0.83 (0.36–1.00)	0.97 (0.82–1.00)	0.36 (0.13–0.65)
** Unwitnessed onset (*n* = 139)**	0.74 (0.65–0.82)	0.80 (0.56–0.94)	0.96 (0.89–0.99)	0.34 (0.21–0.49)

Abbreviation: ASPECTS = Alberta Stroke Program Early CT Score.

#### CT cohort

Alberta Stroke Program Early CT Score was also strongly associated with the presence of a core-perfusion mismatch in the CT cohort: median (IQR) ASPECTS was 8 (6–9) in patients with a mismatch and 5 (2–6) in those without (*P* < .001). As expected, ASPECTS was inversely correlated with ischaemic core volume as defined by relative cerebral blood flow < 30% (Spearman correlation coefficient = −0.69; *P* < .001). The area under the ROC curve for ASPECTS in predicting core-perfusion mismatch was 0.801 (95% CI, 0.698–0.904). The optimal ASPECTS cutoff for classifying core-perfusion mismatch was 7. In patients with ASPECTS ≥ 7, the sensitivity, specificity, positive and negative predictive values were 0.74, 0.81, 0.96 and 0.34, respectively. The diagnostic performance of several ASPECTS cutoffs for identifying core-perfusion mismatch is shown in Table 2 and Figure 1. The results were consistent in the witnessed and unwitnessed stroke-onset subgroups (Table 3). Results were similar in the sensitivity analyses using the alternative core-perfusion mismatch definitions (Tables S2 and S3).

## Discussion

In this large, multicentre international cohort of unselected AIS-LVO patients imaged beyond 4.5 h from last time seen well, we found an association between baseline ASPECTS and the presence of a core-perfusion mismatch, assessed using automated CT- or MR-perfusion imaging. In both imaging modalities, ASPECTS showed good discriminative ability for mismatch identification, with area under the ROC curve values exceeding 0.80.

The diagnostic profiles of ASPECTS differed between MRI and CT cohorts. In the MRI cohort, an ASPECTS ≥ 6 yielded high sensitivity and balanced specificity, with both positive and negative predictive values in the moderate-to-high range (0.84 and 0.77, respectively). This profile suggests that DWI-ASPECTS may be useful for identifying AIS-LVO patients likely to harbour a core-perfusion mismatch, while still retaining reasonable ability to exclude those without. In contrast, in the CT cohort, an ASPECTS ≥ 7 was associated with a very high positive predictive value (0.96) but a low negative predictive value (0.34). This indicates that, beyond 4.5 h, patients with AIS-LVO and high ASPECTS are very likely to have a core-perfusion mismatch, whereas a low ASPECTS does not exclude mismatch. This asymmetry likely reflects the lower sensitivity of noncontrast CT for early infarct core, and the higher overall prevalence of mismatch observed in the CT cohort, which would be expected to increase the positive predictive value and reduce the negative predictive value. The observed variation in ASPECTS threshold performance between modalities may also relate to differences in baseline characteristics between the CT and MRI groups, including age, last-seen-well-to-imaging time and the proportion of witnessed stroke onset.

International guidelines recommend the use of core-perfusion mismatch to guide IVT and EVT beyond 4.5 and 6 h from symptom onset, respectively.^3–5^ However, access to perfusion imaging is limited in some regions or care settings, including primary stroke centres and community hospitals,^10^^,^^11^ underscoring the need for clinical trials aimed at expanding access to these effective therapies for AIS-LVO patients in whom direct assessment of core-perfusion mismatch is unavailable. Our findings suggest that high ASPECTS on CT or MRI could be used as pragmatic enrichment criteria to facilitate patient enrollment in such late-window AIS-LVO trials. With such a design, the majority of enrolled patients would be expected to harbour salvageable tissue and potentially benefit from reperfusion therapy; however, this approach may still inadvertently include approximately 4%–16% of patients without a mismatch, who are unlikely to benefit and may be at increased risk of harm.^6^^,^^7^ Conversely, we found that low ASPECTS values—especially on CT—do not exclude a core-perfusion mismatch and perfusion imaging should be obtained to clarify eligibility for these patients. These findings highlight a fundamental limitation of current imaging paradigms: low ASPECTS does not adequately reflect the extent of potentially salvageable tissue and should not be used as a blanket exclusion criterion. The substantial prevalence of core-perfusion mismatch among patients with low ASPECTS also offers a biologically plausible explanation for the positive late-window subgroup results recently reported in some large-core trials.^21–23^ Nevertheless, TESLA is the only trial using non-contrast CT-only selection in the 24-h time frame, and no EVT treatment benefit was demonstrated in the 6- to 24-h subgroup analysis.^24^ By contrast, the 2 other large-core trials that extended ASPECTS-based selection to 24 h (SELECT2 and ANGEL-ASPECT) required perfusion imaging for all patients, and perfusion findings may have influenced enrolment in these trials.^25^ The proposed ASPECTS thresholds should be viewed as pragmatic approximations for research enrollment rather than as substitutes for perfusion-based treatment selection in routine clinical practice.

The prevalence of core-perfusion mismatch differed substantially between the CT and MRI cohorts (86% vs 62%, respectively). Several factors may account for this. First, differences in clinical characteristics, most notably the higher proportion of unwitnessed stroke onset in the CT cohort. Second, core volume was smaller in the CT cohort, consistent with prior reports showing lower estimated core volumes on CT perfusion compared with DWI when using the relative cerebral blood flow < 30% threshold.^26^ Finally, the volume of tissue with *T*max > 6 s was larger in the CT cohort than in the MRI cohort, despite similar NIHSS scores and occlusion sites. Although one study has suggested comparable *T*max > 6 s volumes between CT and MR perfusion, such comparisons may be software dependent, and direct CT-to-MR *T*max volumes comparisons using RAPID have not yet been performed.^27^ This discrepancy in the prevalence of core-perfusion mismatch across imaging modalities may have important clinical implications for decision-making and warrants further investigation.

To our knowledge, this is the first study to report these findings. Consistent with our results, Desai et al. found that the presence of a clinical-core mismatch was strongly associated with ASPECTS in both the early and late time windows; however, they did not assess the relationship between ASPECTS and core-perfusion mismatch.^28^ By leveraging a large cohort with systematic perfusion imaging across multiple centres and imaging modalities, our study extends this literature by directly quantifying the diagnostic accuracy of ASPECTS for core-perfusion mismatch identification using contemporary, guideline-consistent criteria. Moreover, the inclusion of both treated and untreated patients reduces the risk of selection bias and enhances the generalisability of our findings to real-world stroke patient cohorts.

Our study has limitations. First, ASPECTS assessment, while standardised, remains subject to inter-reader variability, particularly on noncontrast CT.^29^^,^^30^ Although centralised review was performed in most centres, local assessment at 2 sites may have introduced heterogeneity. Second, perfusion imaging was processed using a single software platform, and mismatch definitions were based on established but nonetheless operational thresholds; results may differ with alternative software or definitions. Third, our study focused exclusively on patients with ICA or M1 occlusions. Therefore, the applicability of these findings to other occlusion sites remains uncertain, as the relationship between ASPECTS and core-perfusion mismatch may differ in these populations. Further studies are warranted to determine whether similar findings extend beyond ICA and M1 occlusions. Lastly, this was a retrospective multicentre study involving different time periods and imaging workflows across sites, with image interpretation performed centrally at some sites and locally at others, which may have introduced heterogeneity in patient selection and imaging assessment.

## Conclusion

In a large, multicentre cohort of AIS-LVO patients imaged beyond 4.5 h, ASPECTS was associated with core-perfusion mismatch, with modality-specific thresholds showing good diagnostic performance. The high positive predictive value of ASPECTS ≥ 6 on MRI and ASPECTS ≥ 7 on CT supports their potential use as pragmatic enrichment criteria for patient selection in future late-window AIS-LVO trials when perfusion imaging is unavailable, as surrogate markers of salvageable tissue. Conversely, low ASPECTS values—especially on CT—do not exclude a core-perfusion mismatch and, if available, perfusion imaging should be obtained to clarify eligibility for these patients.

## Supplementary Material

Supplementary_material_aakag065

## Data Availability

The data that support the findings of this study are available from the corresponding author upon reasonable request.
