# Supplementary material for: ASPECTS as a surrogate marker of core-perfusion mismatch in late-window large vessel occlusion stroke
Source: Eur Stroke J. 2026 Jun 8;11(6):aakag065. doi: 10.1093/esj/aakag065 (PMC13245164; doi:10.1093/esj/aakag065)
Supplement: Supplementary_material_aakag065 [file supplementary_material_aakag065.zip › supplemental materials.docx]

**Supplemental materials**

**Supplemental Table 1.** Comparison of key variables between included patients and otherwise eligible patients excluded because of missing or inadequate-quality perfusion imaging

**Supplemental Table 2.** Diagnostic Accuracy of ASPECTS Cutoffs Using An Alternative Core–Perfusion Mismatch Definition (mismatch volume ≥15mL and mismatch ratio ≥1.8 and core volume <70mL)

**Supplemental Table 3.** Diagnostic Accuracy of ASPECTS Cutoffs Using An Alternative Core–Perfusion Mismatch Definition (mismatch volume >10mL and mismatch ratio >1.2 and core volume <70mL)

**Supplemental Table 1. Comparison of key variables between included patients and otherwise eligible patients excluded because of missing or inadequate-quality perfusion imaging**

|  | **Excluded**  **n=68** | **Included**  **n=531** | ***P* value** |
| --- | --- | --- | --- |
| Age, years | 75 (59-81) | 74 (62-84) | 0.56 |
| Male | 29 (43) | 241 (45) | 0.67 |
| NIHSS score | 18 (12-23) | 17 (13-22) | 0.58 |
| Imaging type |  |  | 0.38 |
| MRI | 41 (60) | 349 (66) |  |
| CT | 27 (40) | 182 (34) |  |
| Last seen well to imaging time, hrs | 8.3 (6.0-12.4) | 9.1 (6.3-14.2) | 0.41 |
| Unwitnessed stroke onset | 47 (69) | 325 (61) | 0.21 |
| Occlusion site |  |  | 0.03 |
| ICA | 32 (47) | 177 (33) |  |
| M1 | 36 (53) | 354 (67) |  |
| ASPECT score | 7 (5-9) | 7 (5-8) | 0.16 |
| Thrombectomy performed | 52 (77) | 379 (71) | 0.38 |

Categorical variables are expressed as numbers (%) and continuous variables as median (interquartile range). ASPECT indicates Alberta Stroke Program Early CT score; ICA, intracranial Internal Carotid Artery; M1, first segment of the middle cerebral artery; NIHSS, National Institute of Health Stroke Scale.

**Supplemental Table 2. Diagnostic Accuracy of ASPECTS Cutoffs Using An Alternative Core–Perfusion Mismatch Definition (mismatch volume ≥15mL and mismatch ratio ≥1.8 and core volume <70mL)***

|  | **Sensitivity**  **(95%CI)** | **Specificity**  **(95%CI)** | **Positive**  **predictive value**  **(95%CI)** | **Negative predictive value**  **(95%CI)** |
| --- | --- | --- | --- | --- |
| **MRI cohort** |  |  |  |  |
| ASPECTS ≥ 4 | 0.98 (0.94–0.99) | 0.48 (0.39–0.56) | 0.73 (0.67–0.78) | 0.93 (0.85–0.98) |
| ASPECTS ≥ 5 | 0.95 (0.91–0.98) | 0.60 (0.52–0.68) | 0.77 (0.72–0.82) | 0.90 (0.82–0.95) |
| ASPECTS ≥ 6 | 0.89 (0.84–0.93) | 0.71 (0.62–0.78) | 0.81 (0.76–0.86) | 0.81 (0.73–0.88) |
| ASPECTS ≥ 7 | 0.71 (0.64–0.77) | 0.81 (0.74–0.87) | 0.84 (0.78–0.89) | 0.66 (0.58–0.73) |
| ASPECTS ≥ 8 | 0.48 (0.41–0.55) | 0.87 (0.81–0.92) | 0.84 (0.77–0.91) | 0.54 (0.47–0.60) |
| **CT cohort** |  |  |  |  |
| ASPECTS ≥ 4 | 0.97 (0.93–0.99) | 0.34 (0.20–0.50) | 0.82 (0.75–0.88) | 0.79 (0.54–0.94) |
| ASPECTS ≥ 5 | 0.95 (0.90–0.98) | 0.43 (0.28–0.59) | 0.84 (0.77–0.89) | 0.73 (0.52–0.88) |
| ASPECTS ≥ 6 | 0.90 (0.84–0.94) | 0.70 (0.55–0.83) | 0.91 (0.84–0.95) | 0.69 (0.53–0.82) |
| ASPECTS ≥ 7 | 0.81 (0.74–0.87) | 0.80 (0.65–0.90) | 0.93 (0.86–0.97) | 0.57 (0.44–0.70) |
| ASPECTS ≥ 8 | 0.63 (0.54–0.71) | 0.89 (0.75–0.96) | 0.95 (0.88–0.98) | 0.43 (0.33–0.54) |

* Using this alternative definition, the prevalence of core-perfusion mismatch was 138/182 (76%) in the CT cohort and 206/349 (59%) in the MRI cohort.

**Abbreviations:** ASPECTS, Alberta Stroke Program Early CT score; CI, confidence interval.

**Supplemental Table 3. Diagnostic Accuracy of ASPECTS Cutoffs Using An Alternative Core–Perfusion Mismatch Definition (mismatch volume >10mL and mismatch ratio >1.2 and core volume <70mL)***

|  | **Sensitivity**  **(95%CI)** | **Specificity**  **(95%CI)** | **Positive**  **predictive value**  **(95%CI)** | **Negative predictive value**  **(95%CI)** |
| --- | --- | --- | --- | --- |
| **MRI cohort** |  |  |  |  |
| ASPECTS ≥ 4 | 0.97 (0.94–0.99) | 0.52 (0.43–0.61) | 0.78 (0.72–0.82) | 0.92 (0.83–0.97) |
| ASPECTS ≥ 5 | 0.94 (0.90–0.96) | 0.64 (0.55–0.72) | 0.81 (0.76–0.86) | 0.85 (0.77–0.92) |
| ASPECTS ≥ 6 | 0.87 (0.82–0.91) | 0.74 (0.66–0.82) | 0.85 (0.80–0.90) | 0.77 (0.69–0.84) |
| ASPECTS ≥ 7 | 0.69 (0.63–0.75) | 0.84 (0.76–0.90) | 0.88 (0.82–0.92) | 0.61 (0.54–0.69) |
| ASPECTS ≥ 8 | 0.47 (0.40–0.54) | 0.90 (0.83–0.95) | 0.89 (0.82–0.94) | 0.50 (0.43–0.56) |
| **CT cohort** |  |  |  |  |
| ASPECTS ≥ 4 | 0.96 (0.92–0.99) | 0.33 (0.20–0.50) | 0.83 (0.76–0.88) | 0.74 (0.49–0.91) |
| ASPECTS ≥ 5 | 0.94 (0.89–0.98) | 0.43 (0.28–0.59) | 0.85 (0.78–0.90) | 0.69 (0.48–0.86) |
| ASPECTS ≥ 6 | 0.89 (0.82–0.93) | 0.69 (0.53–0.82) | 0.91 (0.84–0.95) | 0.64 (0.49–0.78) |
| ASPECTS ≥ 7 | 0.80 (0.72–0.86) | 0.79 (0.63–0.90) | 0.93 (0.86–0.97) | 0.54 (0.41–0.67) |
| ASPECTS ≥ 8 | 0.62 (0.54–0.70) | 0.88 (0.74–0.96) | 0.95 (0.88–0.98) | 0.41 (0.31–0.52) |

* Using this alternative definition, the prevalence of core-perfusion mismatch was 140/182 (77%) in the CT cohort and 220/349 (63%) in the MRI cohort.

**Abbreviations:** ASPECTS, Alberta Stroke Program Early CT score; CI, confidence interval.
